# Is nitric oxide a clue to endemic goitre in highlanders?

**DOI:** 10.1186/s13044-023-00183-3

**Published:** 2023-10-02

**Authors:** Jon O. Lundberg, Eddie Weitzberg

**Affiliations:** Department of Physiology and Pharmacology, Karolinska Institutet, Stockholm, Sweden

**Keywords:** Goitre, Nitrate, Nitric oxide, Highlanders, Thyroid, Iodide

## Abstract

Goitre is commonly caused by a lack of iodine in the diet. This condition is particularly prevalent in high-altitude areas where iodine deficiency is common. Here we speculate that inorganic nitrate, the oxidation product of nitric oxide, which is generated endogenously at very high levels in highlanders, further increases the risk of goitre and thyroid dysfunction in this population by inhibiting the transport of iodide into the thyroid gland. Indeed, it is well-known that nitrate and iodide compete for such transport. While iodine scarcity is a primary cause of goitre, the excessive nitrate levels in highlanders may further hinder iodide transport, exacerbating the problem.

Sir,

The causes of goitre are multifold but a lack of iodine (I_2_) in the diet is common worldwide [1]. People living at high altitudes are at greater risk of goitre and prior to the controlled introduction of iodine supplementation through salt, oil and some other vehicles, the incidence in some Himalayan areas could approach 100% in the younger population [2]. Iodide (I^−^), the ionic form of iodine, is an essential element of thyroid hormones and is actively transported into the thyroid gland via a specific transporter, the sodium/iodide symporter, in a process also dependent on ATP, iron and magnesium. It is known that such transport occurs in competition with some other anions including thiocyanate (SCN^−^) and nitrate (NO_3_^−^) [3]. Diets rich in such compounds are sometimes referred to as goitrogenic, as they may lead to a decreased transport of iodide with resulting goitre and thyroid dysfunction [3, 4]. Nitrate is not only found in our diet, it is also generated endogenously being an oxidation product of nitric oxide (NO) [5]. A report published some years ago showed that endogenous NO generation by endothelial NO synthase (eNOS) is exceptionally high in Tibetans [6]. In fact, plasma levels of nitrate in these highlanders are more than 10 times higher than in lowlanders and it was speculated that such massive NO production helps to offset high-altitude hypoxia and to achieve normal oxygen delivery through the vasodilatory effects of NO [6]. We speculate that the persistently high circulating levels of nitrate in highlanders may come with an unwanted side effect, namely inhibition of iodide transport and increased risk of goitre and thyroid dysfunction (Fig. 1). This may help to explain why historically, endemic goitre has classically been seen in high-altitude areas [2]. In addition, the constant exposure to hypoxia may also affect mitochondrial function which could indirectly affect iodide uptake through ATP-dependent processes. While a scarcity of dietary iodine remains the major factor explaining endemic goitre, the additional mechanism discussed here may contribute, in particular in highlanders. In a situation with already little iodine coming from the diet, the endogenously-derived nitrate may become overwhelming, thereby further preventing transport of iodide into the thyroid gland for production of thyroid hormones.

**Fig. 1:**
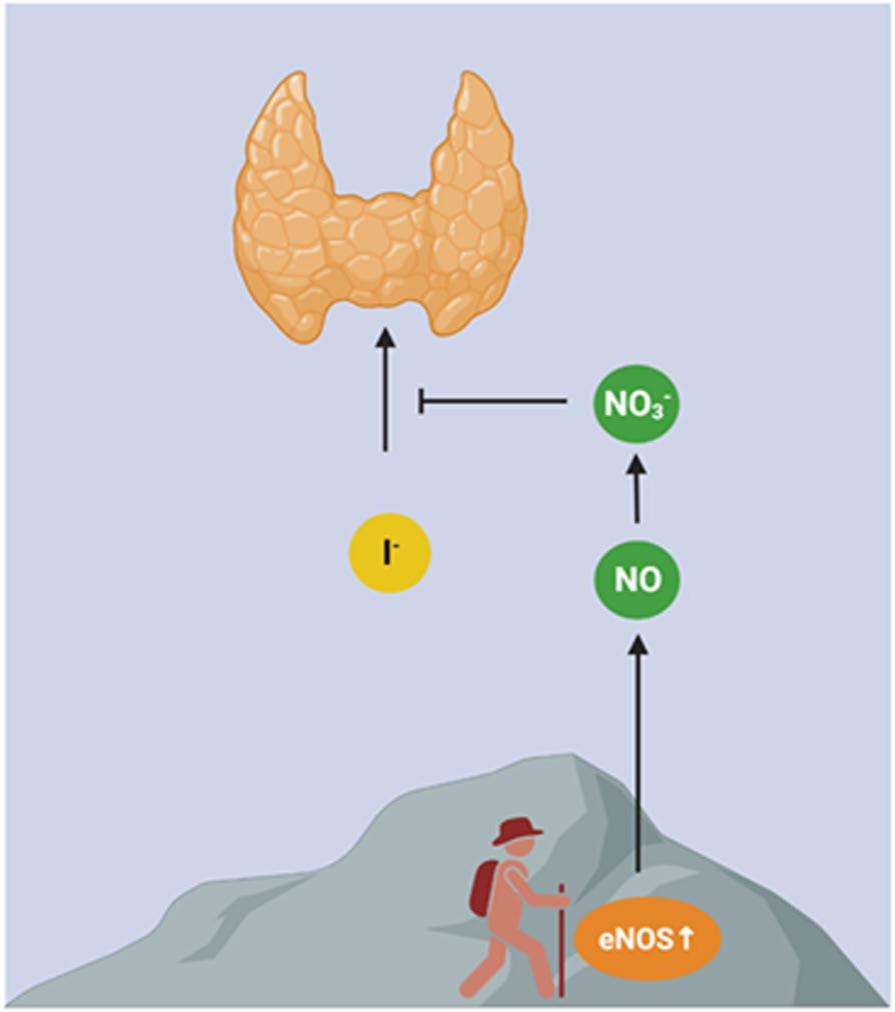
Historically, goitre has been common in highland areas where it could become endemic. This is mainly because the soil is poor in iodine but another mechanism may also be in play. Endogenous generation of nitric oxide (NO) is exceptionally high in highlanders due to continuous high activation of endothelial NO synthase (eNOS) in response to the low oxygen at these altitudes. In the body NO is rapidly converted to nitrate (NO_3_^−^), an anion known to compete with iodide (I^−^) for uptake in the thyroid gland. Here we propose that the high susceptibility to goitre in highlanders may be partly related to the persistently high endogenous formation of NO and nitrate in these populations

## Data Availability

N/A.
